# Editorial: Advances in predicting future adverse coronary events: the role of cardiovascular imaging and coronary physiology indices

**DOI:** 10.3389/fcvm.2023.1206076

**Published:** 2023-05-09

**Authors:** Sara Seitun, Italo Porto, Michail I. Papafaklis

**Affiliations:** ^1^ Department of Radiology, IRCCS Ospedale Policlinico San Martino, Genova, Italy; ^2^ Department of Internal Medicine, University of Genova, Genova, Italy; ^3^ Cardiology Unit, Cardio-Thoracic and Vascular Department, IRCCS Ospedale Policlinico San Martino, Genova, Italy; ^4^ Catheterization and Hemodynamic Unit, Alexandra University Hospital, Athens, Greece

**Keywords:** angiography, computed tomography coronary angiogram (CTCA), intravascular imaging (IVUS), optical coherence tomogaphy, cardiac MRI (CMR), coronary artery disease, acute coronary syndrome (ACS), fractional flow reserve (FFR)

**Editorial on the Research Topic** Advances in predicting future adverse coronary events: the role of cardiovascular imaging and coronary physiology indices

Atherosclerotic coronary artery disease (CAD) remains the leading cause of morbidity and mortality worldwide (1). CAD is the consequence of a multistep and multifactorial process, in which chronic inflammation is involved at every stage. The clinical manifestations of the disease are extremely variable, ranging from subclinical atherosclerosis to plaque progression and acute coronary events. During the last decade, both non-invasive and invasive imaging techniques, as well as assessment of coronary physiology and hemodynamics have provided significant prognostic information. This Research Topic focuses on the prognostic role of cardiovascular imaging and coronary physiology indices for the identification of high-risk patients and coronary lesions. The studies included in this Research Topic address (1) the prognostic implications of non-invasive anatomic and functional imaging techniques (2), new physiologic markers of ischemia (3), new machine-learning assisted analysis of coronary thrombosis (4), the prognostic role of vascular biomarkers in specific subsets of patients, and (5) the advanced diagnostic and prognostic role of invasive intracoronary imaging.

Over the past decade, coronary computed tomography angiography (CCTA) has emerged as a first-line non-invasive imaging modality for the evaluation of patients with suspected CAD, receiving multiple Class 1 recommendations by both the American and European guidelines (2, 3).

The state-of-the-art minireview article by Emfietzoglou et al. addresses the major fields of application of CCTA and future perspectives with new technology such as photon-counting technique with intrinsic spectral capabilities at high contrast and spatial resolution. Besides coronary stenosis assessment, CCTA may evaluate plaque burden and nonobstructive high-risk coronary plaques to predict the risk of future coronary events and to guide targeted treatment and initiation of appropriate preventive therapy. CCTA may also provide the hemodynamic assessment of the coronary lesion via the CT-derived fractional flow reserve (FFR) and myocardial perfusion for guiding treatment decisions. Finally, recent fields of research are the computation of coronary endothelial shear stress, a marker of the initiation and progression of atherosclerosis, and the pericoronary fat attenuation as a sensor of coronary inflammation.

Our series also included several relevant original research articles. The original research by Lee et al. evaluated the prognostic impact of anatomic and hemodynamic plaque characteristics as assessed by CCTA in predicting subsequent coronary events (DESTINY Study). They analyzed 158 patients who underwent CCTA for suspected CAD within 6–36 months before percutaneous coronary intervention (PCI) for acute myocardial infarction (MI) or unstable angina and 62 age- and sex-matched patients without PCI as the control group. They concluded that high-risk plaque characteristics (HRPCs: low attenuation plaque, positive remodeling, napkin-ring sign, spotty calcification, minimal luminal area <4 mm^2^, or plaque burden ≥70%) and hemodynamic parameters [per-vessel FFR (FFRCT), per-lesion ΔFFRCT, and percent ischemic myocardial mass] added incremental prognostic value to clinical factors. Moreover, HRPCs provided more incremental predictability than clinical risk factors alone among vessels with negative FFRCT but not among vessels with positive FFRCT.

Intravascular ultrasound (IVUS) is a useful tool for accurate assessment of coronary lesion complexity and for optimization of stent implantation during PCI. Several meta-analyses of large observational and randomized studies have shown that (IVUS) guidance may improve short and long-term clinical outcomes compared to angiography guidance alone (4–7). However, outside of trials, IVUS use remains highly heterogenous and often low. Tindale et al. analyzed the real-world use and outcomes in PCI-naive patients undergoing IVUS-guided intervention in a specialist heart hospital in the UK. They performed a retrospective analysis of prospectively collected data from 10,574 consecutive patients. Only 4.3% of patients underwent IVUS, with a median follow-up of 4.6 years. There was no difference in survival or major adverse cardiac events (MACE) on both matched and adjusted analysis, although this may well be explained by significant selection bias and the high-risk population. The underuse of IVUS in routine interventional practice is multifactorial, with lack of training, individual operator experience, greater procedural time, and cost barrier being the most reported factors limiting its use (8).

The case report of Krcmar et al. is a great example of how intracoronary imaging with optical coherence tomography (OCT) can guide highly complex procedures, by providing exquisite anatomical details and three-dimensional images. They described a rare and potentially fatal complication of chronic stent loss in the left main and its migration into the left circumflex artery ostium during PCI. This complication was not immediately noticed by the operator, but 5 months later was successfully resolved by OCT guidance with the crushing technique. OCT provided useful information on the exact position and endothelialisation within the undeployed stent.

Coronary flow capacity (CFC) is a relatively new potentially important physiologic marker of ischemia for guiding PCI (9). Hamaya et al. evaluated the determinants and prognostic implications of the changes of thermodilution method-derived CFC status following PCI. In a total of 450 patients with chronic coronary syndrome (CCS), CFC changes following PCI were largely determined by the pre-PCI CFC status and were associated with a lower risk of incident target vessel failure.

Myocardial strain analysis assessed by cardiac magnetic resonance (CMR) feature tracking has emerged as a simple post-processing technique to assess myocardial motion and deformation and has shown to provide additional incremental prognostic value compared to traditional functional parameters in various cardiovascular disease settings (10). Zhuang et al. demonstrated that left ventricular strain and strain rate detected by CMR feature tracking were independent predictors of survival in 78 asymptomatic patients with repaired tetralogy of Fallot who required pulmonary valve replacement.

The original study by Ma et al. analyzed the predictive value of plasma big endothelin-1 (big ET-1) for the occurrence of adverse cardiovascular events in a cohort of patients with in-stent restenosis (ISR) and diabetes mellitus after PCI with drug-eluting stents. Big ET-1 is a 39-amino acid propeptide that is cleaved into the biologically active ET-1. ET-1 is a potent vasoconstrictor, both in large vessels and in the microcirculation, with intramyocardial vessels being very sensitive to its actions. The predictive value of increased circulating ET-1 levels has been shown in most cardiovascular diseases, leading ET-1 to be considered as a likely mediator of excessive vasoconstriction, endothelial dysfunction, cardiac remodeling, and age-associated inflammation (11). At 3-year follow-up, Ma et al demonstrated that big ET-1 improved the predictive value for MACEs over traditional and angiographic risk factors in patients with ISR and diabetes (*n* = 795) but not in non-diabetic patients with ISR (*n* = 998). Therefore, elevated big ET-1 may identify a group of stented patients with diabetes and ISR who are at particular risk for worse outcomes.

ST-segment elevation acute coronary syndrome (STEACS) typically occurs due to occlusive coronary thrombus formation superimposed on a ruptured or eroded atherosclerotic plaque. Kotronias et al. performed an interesting prospective OxAMI (Oxford Acute Myocardial Infarction) study evaluating the pathobiology of coronary thrombosis in patients with STEACS. Thrombus composition has relevant prognostic information since it has been shown that macroscopically red (erythrocyte-rich) thrombi are associated with an adverse prognostic outcome compared to macroscopically white (platelet-rich) thrombi (12). Kotronias et al included 306 patients presenting with STEACS who underwent manual thrombectomy during primary PCI. They used a novel method utilizing spatially resolved reflectance spectroscopy combined with machine-learning approaches for the characterization and quantification of aspirated coronary thrombosis. In a sub-set of 36 patients, they assessed invasive [index of coronary microvascular resistance (IMR)] and non-invasive [microvascular obstruction (MVO) at CMR] indices of coronary microvascular injury. They demonstrated that this novel analytical approach enabled the quantification of thrombus composition, including automated quantification of thrombus area within the images. Finally, the derived reflectance spectral signatures of coronary thrombi were correlated with microvascular injury phenotyping, as measured by MVO and IMR.

In conclusion, the articles included in this Research Topic are small but important steps in our understanding of CAD, adding new knowledge about the prognostic role of different imaging techniques, coronary physiology indices, and cardiovascular biomarkers. In the coming years, it is expected that further work could provide a deeper insight into the pathophysiology of coronary atherosclerotic process with the ultimate aim to improve patient outcomes, reduce the burden of cardiovascular disease and aid in the development of new therapeutic strategies.
